# Detrimental Effects of Microgravity on Mouse Preimplantation Development In Vitro

**DOI:** 10.1371/journal.pone.0006753

**Published:** 2009-08-25

**Authors:** Sayaka Wakayama, Yumi Kawahara, Chong Li, Kazuo Yamagata, Louis Yuge, Teruhiko Wakayama

**Affiliations:** 1 Laboratory for Genomic Reprogramming, RIKEN, Center for Developmental Biology, Kobe, Japan; 2 Division of Bio-Environment Adaptation Sciences, Graduate School of Health Sciences, and Space Bio-Laboratories, Hiroshima University, Hiroshima, Japan; 3 Department of Bioscience, Graduate School of Science and Technology, Kwansei Gakuin University, Sanda, Japan; Cincinnati Children's Research Foundation, United States of America

## Abstract

Sustaining life beyond Earth either on space stations or on other planets will require a clear understanding of how the space environment affects key phases of mammalian reproduction. However, because of the difficulty of doing such experiments in mammals, most studies of reproduction in space have been carried out with other taxa, such as sea urchins, fish, amphibians or birds. Here, we studied the possibility of mammalian fertilization and preimplantation development under microgravity (µG) conditions using a three-dimensional (3D) clinostat, which faithfully simulates 10^–3^ G using 3D rotation. Fertilization occurred normally in vitro under µG. However, although we obtained 75 healthy offspring from µG-fertilized and -cultured embryos after transfer to recipient females, the birth rate was lower than among the 1G controls. Immunostaining demonstrated that in vitro culture under µG caused slower development and fewer trophectoderm cells than in 1G controls but did not affect polarization of the blastocyst. These results suggest for the first time that fertilization can occur normally under µG environment in a mammal, but normal preimplantation embryo development might require 1G.

## Introduction

Changes in the gravitational field have significant effects on the development of plants and animals [1]. Therefore, the potential effect of a microgravity (µG) environment on reproduction has been a major biological theme in the age of space exploration. So far, several experiments on reproduction in such environments have been reported using sea urchins, fish, amphibians and birds, and the fertilization rates were similar to those found in controls at normal gravity (1G) [1]–[7]. However, unlike the other taxa studied to date, mammalian reproduction is complicated and highly specialized. Oocytes do not have enough resources to support full term development, so after fertilization, the embryo needs to implant in the uterus and to be supplied from the mother via the placenta. Studies on rats have shown that µG affected reproduction: there were decreased total sperm numbers [8], increases in sperm abnormalities [9] and reduced testicular weights during space flight [10], [11]. In the STS-80 space shuttle mission, mouse 2-cell embryos were collected on the ground, launched into space and cultured for four days in µG. The control embryos on Earth developed to normal blastocysts, but in the space flight group, none of the embryos showed any sign of development, and all degenerated [12]. A more reliable experiment was done on the Cosmos 1129 mission in 1979, when mature male and female rats were sent into orbit and then allowed to intermingle in a common breeding chamber [13]. However, none of the females gave birth, although postflight examinations revealed that ovulation had occurred. Two of the females were reported to have achieved pregnancy, but the embryos appear to have been resorbed. Although this experiment did not examine whether fertilization or preimplantation development occurred normally, this raised the important question of whether mammalian reproduction is indeed possible in space. However, further such experiments have not been performed so far because of technical difficulties in using live animals. Mammalian reproduction is very sensitive to environmental factors. For example, with mice and rats, if the breeding room is changed, the estrous cycle is altered, the number of oocytes ovulated is reduced and mating can fail [14]. If mice were to be taken into space, they would be exposed to strong vibrations and hypergravity during the launch, and then suddenly exposed to the additional stress of µG conditions. In these situations, it is highly unlikely that the mice would copulate during the flight period. Actually, in the Cosmos 1129 mission, there were no pregnancies even among the ground-based 1G controls [15].

In vitro fertilization (IVF) might solve this problem if we could launch oocytes and sperm into space instead of live animals. On the ground, mouse IVF is now well established [16], [17]. However, to perform IVF in space, we must develop several new techniques. For example, oocytes lose their fertilizability soon after ovulation [18]; therefore, it is impossible to collect oocytes from animals and preserve them for use in space environments before launch. Oocyte and sperm cryopreservation could be used to store the gametes for IVF in space, but oocyte freezing is still imperfect [19]. Moreover, IVF is a difficult biological procedure, and fertilization can fail even in a ground-based laboratory if the experimentalist is inexperienced.

Methods for simulating µG on Earth have been investigated as alternatives to expensive space flight. A microgravity condition can be produced either by a space flight or by a free fall. However, the duration of a microgravity condition produced by a free fall is usually too short to alter cell growth and differentiation. Because of limited access to space flight, many efforts have been made to establish alternative methods for simulating microgravity on Earth, and one of these—the ‘clinostat’—is considered to be a suitable device. The clinostat mimics µG by ‘nullifying the gravitational vector’ through continuous averaging of G forces using three-dimensional (3D) rotation (see Movie S1) [20]. Moreover, this device can subject the specimen to µG conditions immediately, without exposure to the hypergravity and strong vibrations of a space launch. This allows the investigator to test the effects of µG conditions in isolation from other physical factors.

In earlier studies, the clinostat was rotated only in a single dimension (1D), such as horizontal rotation [21] or vertical rotation [22], [23], which generated extra centrifugal forces and made it difficult to nullify the gravitation vector fully. Although the USA's National Aeronautics and Space Administration (NASA) has improved this system [24], the results of some papers have been contradictory [22], [25]. Recently, a 3D clinostat has been developed to generate a multidirectional G force, resulting in a more accurate environment averaging 10^–3^ G [25]–[27], which is the same as inside a space shuttle (Movie S1). Several studies of cultured cells have been published using a 3D clinostat [28], and the results were similar to true space experiments [29], which validated its effectiveness for simulation [30]. Therefore, 3D clinostat experiments will help in predicting and planning real space experiments, which can reduce costs and effort.

Interestingly, one recent study discovered that the µG condition generated by a 3D clinostat inhibits the differentiation of stem cells [25]. Thus, a 3D clinostat might have clinical applicability in regenerative medicine to help proliferate stem cells for patients. However, if this effect is also true for undifferentiated early embryos, they might fail to differentiate or develop to offspring.

In the present study, we examined in vitro fertilization and preimplantation development in the mouse under µG using a 3D clinostat. To evaluate the embryos, some were examined for quality and cell numbers by immunostaining, and others were transferred into pseudopregnant recipient females to test their potential for development to full term.

## Results

### Effects of µG on in vitro fertilization (IVF)

**Figure 1:**
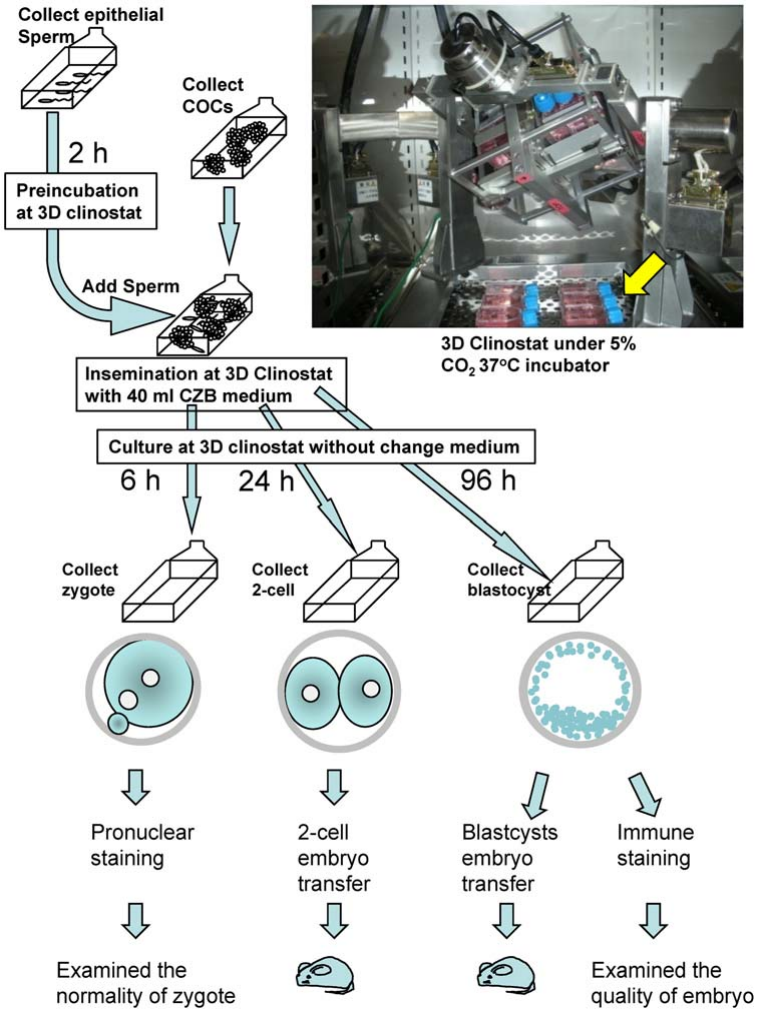
Experimental procedure and 3D clinostat.

**Figure 2:**
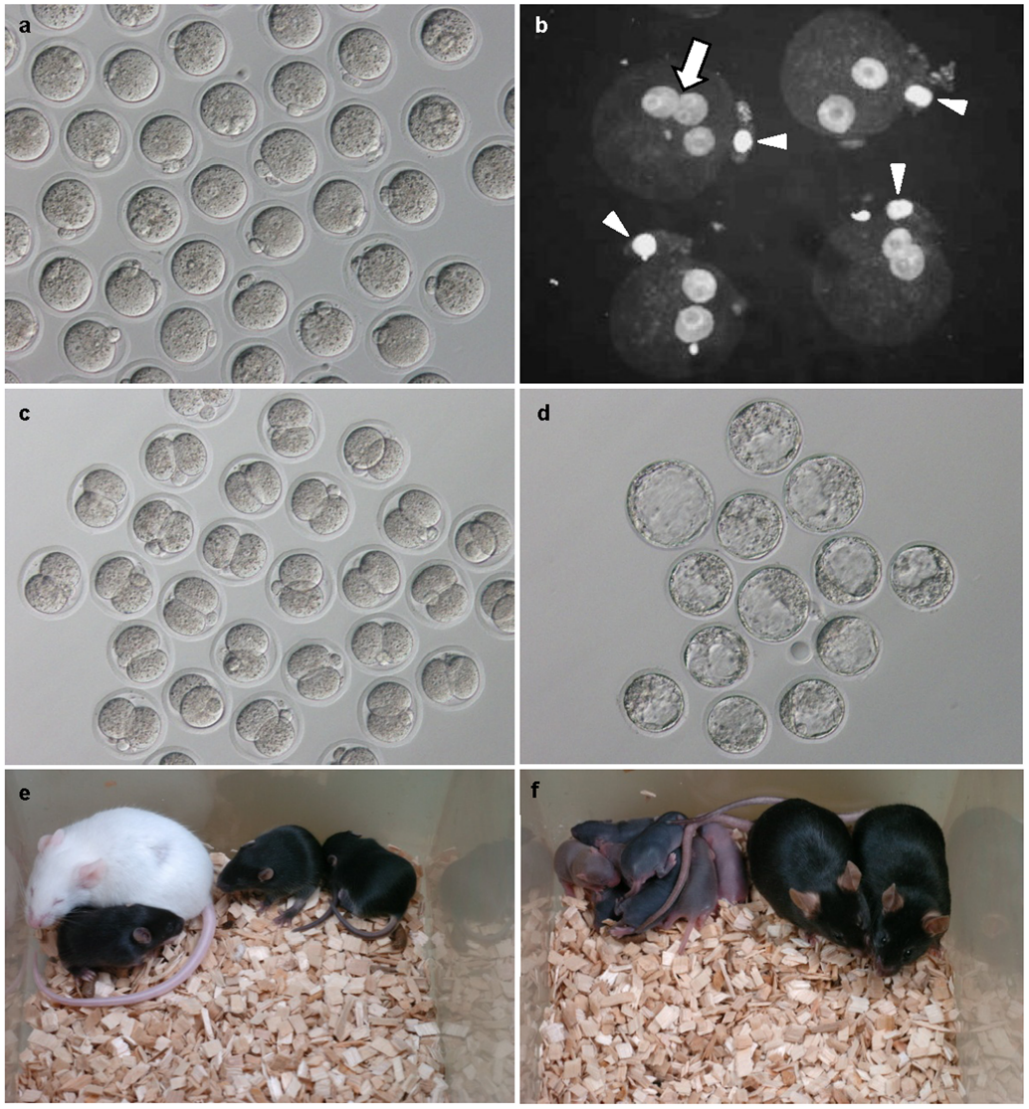
Preimplantation development of microgravity (µG)-fertilized and -cultured embryos and development of full-term offspring. Mouse oocytes were fertilized with spermatozoa preincubated under µG. (a) Zygote at 6 h after insemination. (b) Nuclear staining with DAPI. The arrow indicates a polyspermic (3 PN) fertilized zygote, and the arrowhead indicates a second polar body. (c) Two-cell stage embryo cultured for 24 h under µG. (d) Blastocyst cultured for 96 h under µG. (e) Offspring derived from µG-fertilized and -cultured blastocysts. Two to three months later, these offspring grew to adulthood, and randomly selected mice were proven fertile in natural mating (f).

At 6 h after IVF, the clinostat was stopped, and zygotes were collected from the culture flasks. Control studies at 1G were performed using the same batch of preincubated spermatozoa and the same incubator but outside the clinostat (Fig. 1a, arrow). The fertilization rate, judged by second polar body extrusion, was very similar between 1G and µG (Fig. 2a, Table 1). We also confirmed the normality of zygotes by pronuclear (PN) staining (Fig. 2b). In µG conditions, although polyspermic 3-PN zygotes were significantly more frequent than controls, there was a lower rate of parthenogenetically activated 1-PN zygotes. This suggests that mouse sperm motility might be enhanced slightly under µG. Similar phenomena were observed in actual space experiments using sea urchin and bull spermatozoa [31], [32]. However, most of these oocytes (84%) were fertilized normally, judged by second polar body extrusion and the presence of 2 PN: similar to 1G controls (80%; Table 1). Therefore, µG appears to have no harmful effects on mouse gametes—at least in terms of fertilization in vitro.

**Table 1:** In vitro fertilization of mouse gametes under microgravity.

Category	Collected oocytes	Fertilized (total)	Parthenogenetic (%)	Polyspermic (%)	Normal (%)
1G	203	184	33 (18)	2 (1)	149 (81)
µG	229	207	8 (4)	25 (12)	174 (84)
*p*-Value			>0.01	>0.01	<0.01

### Effects of µG on development in vivo

To demonstrate the normality of µG fertilized and cultured embryos, the strongest evidence is to show the potential to develop to full-term offspring. Therefore, we transferred µG-generated embryos at either the 2-cell stage (24 h culture) or the blastocyst stage (96 h culture) to oviduct of 0.5 dpc or uteri of 2.5 dpc recipient pseudopregnant females, respectively. In this experiment, to mimic the µG condition exactly, embryos were cultured continuously in the clinostat from IVF to the time of embryo transfer. Therefore, we could not determine the exact fertilization or embryo development rates in each flask. However, control 1G experiments were performed under exactly the same conditions (Fig. 1a) except that they were outside the clinostat.

As shown in Table 2, when embryos were collected from µG-cultured flasks at 24 h, more than half of the embryos had developed to the 2-cell stage (Fig. 2c), without any difference from controls. However, after transfer to recipient females, the rate of production of offspring from µG-cultured embryos (35%) was significantly less than with the 1G controls (63%). When embryos were collected from the µG culture flask at 96 h, the rate of development to the blastocyst stage (30%; Fig. 2d) was also significantly lower than control cultures (57%). It should be noted that these control results are poorer than with conventional IVF methodology [16]–[18], [33]–[37]. As discussed above, the use of a flask and a large volume of medium were essential in using the 3D clinostat system, and our preliminary experiments showed that these had negative effects on embryo development. However, the µG experiments and control studies were done at exactly the same time and under the same conditions, except for the use of the 3D clinostat (Fig. 1a, arrow). Therefore, we believe that our results are valid.

**Table 2:** In vitro development of embryos under microgravity.

Culture period	Category.	*p*-Value	Collected embryos	In vitro development (%)		
				No. abnormal	No. 2-cell	No. blastocysts
24 h	1G	<0.05	51	24	27 (53)	–
	µG		494	230	264 (53)	–
96 h	1G	>0.05	42	18	–	24 (57)
	µG		282	196	–	86 (30)

After transferring blastocysts to recipient females (see Table 3), the rate of producing live offspring from µG-cultured embryos (16%; Fig. 2e) was also significantly lower than with the 1G controls (37%). Although fewer offspring resulted from µG culture than in the 1G controls, their body and placental weights were within normal range, and the mice grew to adulthood. Three male and three female mice derived from the µG-cultured blastocyst transfers were selected at random and paired for mating. All pairs delivered litters a few months later (Fig. 2f), demonstrating that the fertility of µG-generated offspring was normal.

**Table 3:** Production of offspring from embryos fertilized and cultured under microgravity.

Culture period	Category.	*p*-Value	Embryo transfer	No. pups (%)	Weight (g)±SD	
					Pups	Placenta
24 h (2-cell)	1G	>0.05	27	17 (63)	1.49±0.13	0.11±0.02
	µG		172	61 (35)	1.40±0.11	0.11±0.02
96 h (Blast)	1G	>0.05	24	9 (38)	1.46±0.15	0.13±0.02
	µG		86	14 (16)	1.41±0.12	0.12±0.03

### Effect of µG for cell differentiation and polarization of blastocyst

**Figure 3:**
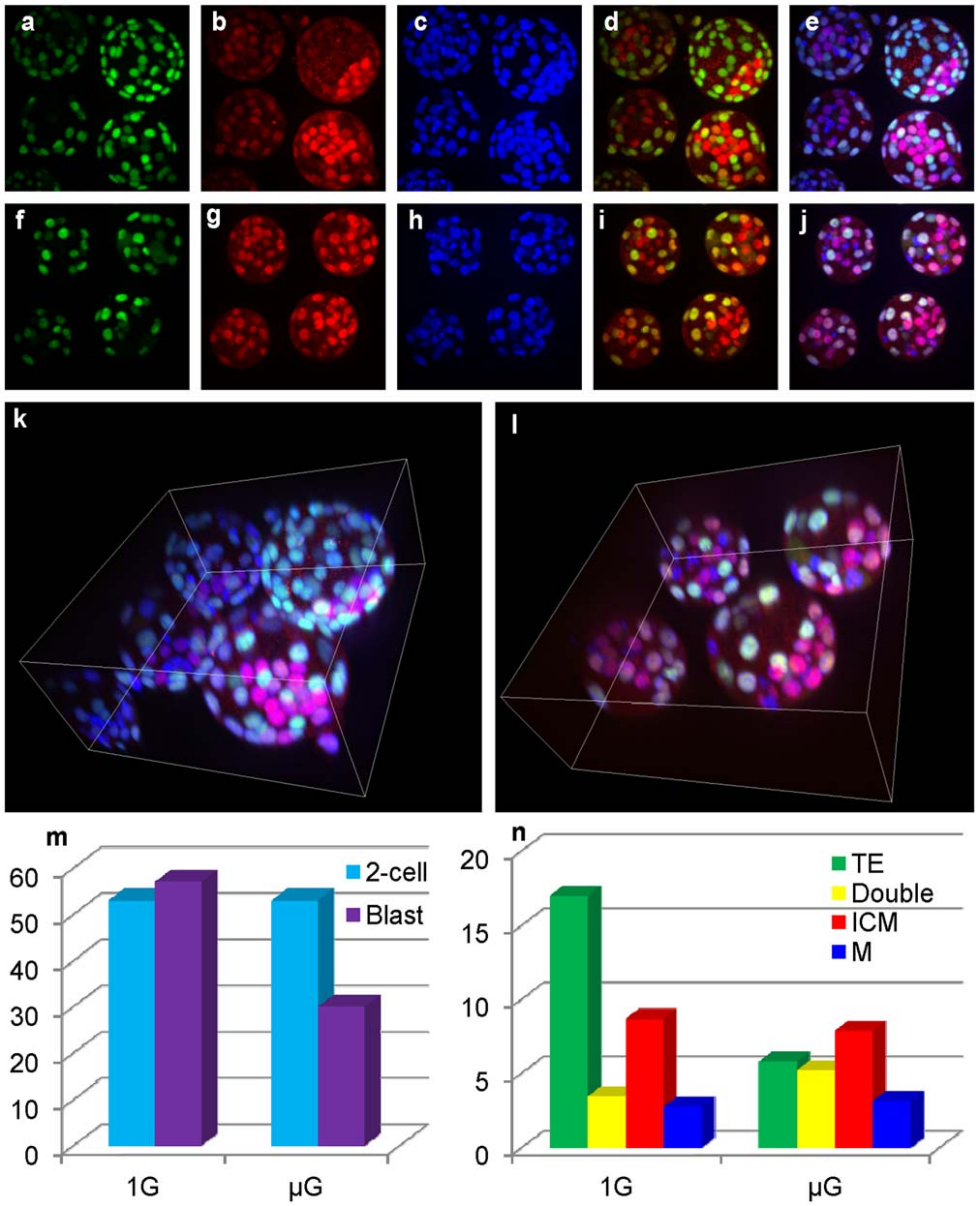
Blastocyst quality after culture under microgravity. The trophectoderm (TE) and inner cell mass (ICM) cell numbers were counted by immunostaining for Cdx2 (a and f, green) or Oct4 (b and g, red), respectively. DNA was stained with DAPI (c and h, blue). Cdx2/Oct4 doubly positive cells were counted on merged images (d and i). Metaphase cells (DAPI staining only) were counted on triple-merged images (e and j). The ICM localization was examined using a three-dimensional (3D) viewer (k and l, and Movie S2 and S3). As we acquired 51 focal planes in the *z*-axis, we could determine the 3D structure of all embryos. (m) Rates of development to the 2-cell and blastocyst stages in 1G and µG culture systems. (n) Cell numbers and cell types compared between 1G- and µG-cultured blastocysts.

As shown in Table 2 and Table 3, 96 h culture in µG conditions caused a reduced rate of development to the blastocyst stage, as well as reduced full-term development following transfer. To examine the reason for this low rate of development in µG conditions, we examined the quality of blastocysts based on cell number (Table 4), cell differentiation and polarity, using immunostaining (Fig. 3). In mice, the transcription factor Oct4 is expressed throughout preimplantation development and is restricted to the inner cell mass (ICM) at the blastocyst stage [38]–[43]. Oct4 is required for the maintenance of ICM fate and the pluripotency of ES cells. Trophectoderm (TE) differentiation begins before the downregulation of Oct4 in the outer cells and is marked by Cdx2 expression. Therefore, only Cdx2-positive cells were judged as differentiated, and only Oct4 positive cells were judged as undifferentiated. Oct4/Cdx2 double-positive cells were judged as transition stage from undifferentiated to differentiated. Nonreactive cells that were DAPI positive (nuclear staining) were judged to be in metaphase.

**Table 4:** Cell numbers and populations in microgravity-fertilized and cultured blastocysts.

Category.	Total cell number	No. TE cells (%)	No. transition (%)	No. ICM cells (%)	No. M phase cells (%)
		(Cdx2 positive)	(double positive)	(Oct4 positive)	(DAPI only)
1G	32.0±10.4	17.0±10.6 (53)	3.5±2.7 (11)	8.7±3.4 (27)	2.8±2.0 (9)
µG	22.3±9.3	5.9±5.6 (26)	5.3±2.5 (24)	8.0±3.0 (36)	3.2±3.2 (14)
*p*-Value	>0.05	>0.05	>0.05	<0.05	<0.05

We examined 21 of the µG-cultured blastocysts and 26 control 1G blastocysts. The mean ICM cell number in µG blastocysts (8.0 cells) was almost same as in the 1G controls (8.7 cells; Fig. 3d, i; red). However, downregulation of Oct4 was incomplete in the µG blastocysts, and the numbers of cells that were doubly positive for Oct4 and Cdx2 were increased significantly (Fig. 3d, i; yellow). There were fewer completely differentiated TE cells in µG-cultured blastocysts (5.9 cells) than in 1G controls (17.0 cells) (Fig. 3d, i; green). In addition to cell number, we also observed the localization of the ICM in blastocysts using a 3D imaging system (Fig. 3k and l and Movie S2 and S3). If ground-level gravity is essential for polarity in the blastocyst, the localization of the ICM in µG-cultured blastocysts might be impaired, and this could allow Oct4-positive cells to disperse into the blastocoel. However, the ICM was located normally in all the µG-cultured blastocysts. These results suggest that µG culture conditions impaired both the rate of embryo growth to the blastocyst and differentiation into the TE lineage. Nevertheless, polarization of the ICM and TE components was unaffected.

## Discussion

In this study, we produced healthy offspring from embryos produced under µG conditions including sperm preincubation, IVF and in vitro culture to the blastocyst stage. IVF occurred normally under µG conditions. However, the quality of blastocysts grown under µG conditions was reduced.

The production of healthy offspring is the strongest evidence of the normality of µG-fertilized embryos. At 24 h after IVF, although live offspring were born after embryo transfer into recipient females, the overall rate of production from the µG embryos was significantly lower than in the 1G controls (p<0.05). Moreover, when culture periods were extended to 96 h in µG, the live birth rate was significantly lower than in controls (5% vs. 21%). Kojima et al. reported similar results using a 1D clinostat [44]. However, they showed no difference in offspring production rates between µG and 1G. Possibly this reflects differences between the 1D and 3D clinostat environments. Another possibility for the discrepancy is that they transferred zygotes (6 h after IVF) into recipient females, whereas we transferred 2-cell embryos (24 h after IVF), which reinforces the idea that prolonged µG culture impairs embryo quality.

These results strongly suggest that although fertilization had occurred normally, µG conditions had a harmful effect on embryo development even after one day of exposure. If we could remove the negative effects of a large volume of medium, for example by using a completely self-contained microfluidics chamber, this could facilitate study on the real effects of µG.

In this study, 61 and 14 pups were obtained from embryos cultured for 24 h or 96 h under µG conditions, respectively. All these offspring appeared normal, and randomly selected animals were later proven fertile by natural mating. This suggests that, although embryo growth was impaired under µG conditions, some of the treated embryos maintained a normal potential for development. However, obviously the recipient females could not be kept in the clinostat, so implantation and gestation in vivo was at 1G. The µG-fertilized and -cultured embryos would thus have spent about 2 days (2-cell embryo transfer) or half a day (blastocyst transfer) in female body under 1G conditions before implantation. It is possible that even if all the µG-fertilized and -cultured embryos were abnormal, this was corrected in some embryos before implantation.

To test the effect of prolonged µG culture, we examined the quality of blastocysts by immunostaining at 96 h after IVF. The TE cell numbers in µG-cultured blastocysts were significantly lower than in the 1G controls, whereas the numbers of other cell types were unaffected (Fig. 3n). There are two possible explanations. One is that µG conditions slow embryo growth rates, another is that they affect the potential for cell differentiation. It was reported that the µG conditions generated by a 3D clinostat inhibited stem cell differentiation [25]. However, it is unclear whether this also affects mammalian embryos. However, if so, µG conditions appear to inhibit the differentiation of TE cells from totipotent blastomeres, rather than slowing embryo growth. On the other hand, the localization of the ICM in µG-cultured blastocysts appeared normal. In amphibians, in which the zygotes show strong polarity during early cell division, µG had no harmful effect on embryo development [45]. Thus, the effects of µG might be limited to embryonic cell growth rate or differentiation, but µG does not appear to impair cell localization within the blastocyst. In other words, the polarization of preimplantation embryos was independent of gravity in this model.

Unlike studies on early development, far more space research has focused on the latter half of pregnancy, during which many of the early, sensitive phases of development including placentation and early organogenesis normally occur at 1G. Thus far, female rats at several different stages of pregnancy have been launched into space. These data provide clear evidence that rats kept in space at µG during the latter half of their pregnancies are able to sustain normal body weight gains and to support the growth and development of their gestating offspring [46]–[50]. These results were consistent with those for other species, especially urodeles [51] and fish [3], [52]. For example, salamander and Medaka fish eggs can be fertilized and develop normally during orbital flight [2], [45]. Those results and ours combined suggest that µG conditions do not affect fertilization or later fetal development. However, µG impairs the development of preimplantation-stage mammalian embryos and possibly implantation as well. It will be very important to know whether implantation can occur in space under µG conditions. This question could be addressed when the Japanese Experiment Module ‘Kibo’ of the International Space Station (ISS) starts appropriate experiments.

## Materials and Methods

### Animals

B6D2F1 (C57BL/6J×DBA/2) mice were used as sources of oocytes and spermatozoa. ICR (CD-1) strain mice were used as pseudopregnant recipient. Both strains were obtained at 8–10 weeks of age from Japan SLC, Inc. (Hamamatsu, Japan). All animals were maintained in accordance with the Animal Experiment Handbook at the Center for Developmental Biology, RIKEN, Kobe, Japan. Female mice were induced to superovulate with consecutive injections of equine chorionic gonadotropin (5 IU) and human CG (hCG; 5 IU) 48 h apart. Fourteen hours after the hCG injection, mice were killed to collect oocytes.

#### Ethics Statement

The protocols for animal handling and treatment were reviewed and approved by the Animal Care and Use Committee at the same institution.

### Adaptation of IVF to the 3D clinostat system

In the conventional IVF protocol at 1G, mouse sperm are preincubated in vitro with 400 µl of IVF medium for 1–2 h in an incubator at 37°C to develop their fertilization potential (capacitation) [16] and then capacitated spermatozoa are transferred into another 400 µl of IVF medium containing oocytes. After 6 h culture, fertilized zygotes are collected, washed and then zygotes are cultured in a different dish with 20–50 µl of embryo culture medium. About 72 h after insemination, embryos that develop to morula/blastocyst (day 4) were transferred into day 3 pseudo-pregnant females to give live offspring.

On the other hand, the 3D clinostat system required us to use 12.5 cm^2^ flasks filled (Falcon #353107, with a filter cap) with about 40 ml of medium: 100 times more than in conventional IVF. In addition, to maintain µG conditions completely, embryos were cultured in the same flask until the blastocyst stage without washing. In this situation, the culture volume is 1000 times greater than with the conventional culture system. Thus, before starting experiments, we had examined the effects of medium volume, of different media, and prolonged culture in a flask with continuous exposure to spermatozoa in terms of embryo development.

In preliminary IVF experiments, we examined the effect of µG on sperm preincubation, and there was no difference from control studies (data not shown). Therefore, we used spermatozoa capacitated in µG conditions for all experiments. Although sperm were preincubated in IVF medium (TYH; Mitsubishi Kagaku, Tokyo) [16], insemination was performed in embryo culture medium (CZB) [53] rather than TYH medium, because the zygotes needed to be cultured continuously under µG conditions without a wash step. However, the fertilization rate with this system was almost the same as with conventional IVF (91% and 88%, respectively; repeated three times). This result suggests that IVF can be achieved without any problem in embryo culture medium rather than IVF medium, and that the large amount of medium in the µG flask had no effect on this outcome.

In terms of extended culture, we have compared the rate of blastocyst development in the flask culture method with the standard protocol outlined above. At 96 h after IVF, the rate of blastocyst development was significantly decreased in flask cultures compared with routine dish cultures (63% vs 97%, respectively; repeated three times). Although a low embryo-to-culture volume ratio is critical for the optimal development of embryos in vitro [54]–[56]it would be impossible to produce sufficient numbers of embryos to compensate for the 1000-fold greater volume of flasks compared with microdroplets in dishes. This suggests that a decrease in the rate of blastocyst development is inevitable when using a relatively large flask. However, even if the large volume of medium affects embryo development, in this study the 1G controls and µG experiments were performed in exactly the same conditions, except for the use of the 3D clinostat (see Fig. 1 arrow). Therefore, this appropriate control enabled us to identify the exact effects of µG during IVF and embryo development.

In terms of embryo transfer, in the standard protocol, in vitro-produced/manipulated embryos are transferred into 1-day-delayed pseudopregnant females to compensate for the timing of development. Therefore, 2-cell-stage embryos at 24 h after insemination (day 2), or morulae/blastocysts at 72 h after insemination (day 4) are normally transferred into the oviduct (day 1) or uterus (day 3), respectively. However, we noticed that when embryos were cultured in flasks, blastocyst development was retarded and the embryos required 5 days to develop to the expanded stage. In addition, it was known that the developmental potential of offspring was unaltered when embryos were cultured in vitro for 4 or 5 days. However, when embryos were cultured for 6 days, the implantation rates of transferred embryos decreased significantly [57]–[59]. Therefore, in this study, 2-cell embryos were transferred into 1-day-delayed recipient females as usual. However, day 5 blastocysts were transferred into 2-day-delayed (day 3) recipient females. Although the rate of producing offspring after embryo transfer was lower than with the ordinary method (29% vs 57%, respectively; repeated three times), this modified method was entirely reproducible.

### Sperm and oocyte preparation

Mouse IVF medium (TYH) was added to the flask one day before experiments and equilibrated in a 37°C, 5% CO_2_ incubator. Next day, epididymides were collected from two euthanized male mice. A dense sperm mass was squeezed out from the cut epididymis and placed into the flask. TYH medium was added to fill the flask without any bubbles. The flask was capped and placed in the 3D clinostat, which was then started. During sperm preincubation, oocyte cumulus complex (COCs) were collected from the oviducts of two or three females, and transferred into a Falcon flask filled with CZB medium that had been equilibrated for one day before use and then placed in the incubator until starting the experiment. Because COCs were used to enable high fertilization rates, we could not count the number of oocytes in each flask. However, after the experiments, we found that each flask had 60–100 oocytes.

### In vitro fertilization

After finish the sperm preincubation, the flasks were removed from the 3D clinostat, and 5 ml aliquots of sperm suspension were introduced into the flasks containing the oocytes. The average final sperm concentration of this method was about 10^6^/ml. CZB medium was added to fill the flask without any bubbles. The 3D clinostat was then started immediately. It took less than 20 min from collecting the sperm suspension to restart the 3D clinostat for IVF. In control experiments, IVF was performed under exactly the same conditions without using the 3D clinostat, and gametes were cultured in the same incubator at the same time (Fig. 1).

### Examination of µG-fertilized zygotes

At 6 h after IVF, all oocytes were collected from the flasks and the fertilization rates were estimated, based on second polar body extrusion. Then all zygotes were fixed in 4% paraformaldehyde in phosphate buffered saline (PBS) for 30 min, washed twice with 1% bovine serum albumin (BSA) in PBS, transferred into 1% BSA–PBS containing 0.1% Triton X-100 (Nacalai Tesque Inc., Kyoto, Japan) and incubated overnight at 4°C. All zygotes were washed, and their DNA was stained with 6-diamidino-2-phenylindole (DAPI) (2 µg/mL; Molecular Probes Inc., Eugene, OR, USA). Some zygotes were incubated with a rabbit polyclonal antibody directed against histone H3 trimethyl K9 (ChIP grade; ab8898, Abcam plc, Cambridge, USA) at room temperature for 2 h before DNA staining. After the embryos had been washed twice in 1% BSA–PBS for 15 min each, they were incubated for 1 h with the fluorochrome-conjugated secondary antibody, Alexa Fluor 488-labeled chicken anti-rabbit IgG (Molecular Probes Inc.). This staining protocol is specific for the female PN. All the embryos were observed using a confocal scanning laser microscope (FV-1000, Olympus, Tokyo, Japan).

### Embryo transfer

At 24 h or 96 h after IVF, all embryos or oocytes were collected from the flasks. The rates of 2-cell or blastocyst development were calculated from all collected oocytes, including abnormally ovulated ones (these cannot be distinguished from unfertilized oocytes at this stage). Two-cell stage embryos were transferred to oviduct of pseudopregnant ICR mice at 0.5 days post copulation (dpc). These had been mated with a vasectomized ICR male the night before transfer. Blastocyst-stage embryos were transferred to the uteri of pseudopregnant mice at 2.5 (dpc. Six to 10 embryos were transferred into each oviduct or uterus, respectively [17], [33], [37]. At days 18.5 to 19.5 dpc, the offspring were delivered naturally or by cesarean section, and we recorded the body and placental weights and sex. All offspring were fostered to other females to allow them to grow to adulthood. When they matured sexually, randomly selected males and females (three each) were paired and mated to confirm their fertility.

Note that the 3D clinostat culture was carried out in Hiroshima, and the animals were maintained in Kobe. Therefore, after finishing each experiment, the 1G control and µG culture flasks were moved from Hiroshima to Kobe, which takes 3 h, but the flasks were kept at 37°C in a warming box. As shown in the 1G control experiments, this short travel did not affect embryo development.

### Assessment of blastocyst quality by immunostaining

In preliminary experiments, we found that embryo development was slightly slower than normal in both µG and control 1G conditions when this modified IVF system was used. Therefore, in this study, we examined the quality of blastocysts at day 4.5 of culture (96 h after IVF).

In the 96 h experiments, all the embryos were fixed immediately after stopping the clinostat. The total cell numbers of blastocysts were counted, and localizations of the ICM and TE cells were observed by immunostaining. Briefly, blastocysts were fixed in 4% paraformaldehyde in PBS for 40 min at room temperature, washed twice with PBS containing 0.5% polyvinylpyrrolidone (Sigma-Aldrich, St Louis, MO, USA) and permeabilized with PBS containing 0.25% Triton X-100 (Nacalai Tesque, Inc.) for 20 min at room temperature (RT). After blocking with 1% BSA/PBS containing 0.1% Tween-20 (Sigma-Aldrich) for 1 h, the blastocysts were further incubated with the primary antibody diluted in the blocking solution at RT for 2 h. The antibodies used were anti-POU5F1/Oct3/4 rabbit polyclonal antibody (1∶200; Santa Cruz Biotechnology, Inc., Santa Cruz, CA, USA) for detecting the ICM, and anti-CDX2 mouse monoclonal antibody (1∶200; BioGenex, Inc., San Ramon, CA, USA) for detecting the TE. After the embryos had been washed twice in the blocking solution, they were incubated for 1 h with fluorochrome-conjugated secondary antibodies: Alexa Fluor 488-labeled goat anti-rabbit IgG (Molecular Probes, Inc.) or Alexa Fluor 568-labeled goat anti-mouse IgG (Molecular Probes, Inc.). The embryos were washed again, and their DNA was stained with 4′,6-diamidino-2-phenylindole (DAPI) (2 µg/mL; Molecular Probes, Inc.).

To analyze the embryos three-dimensionally, the embryos were transferred to 5 µl drops of 1% BSA–PBS in a glass-bottomed dish and observed under an inverted fluorescent microscope (IX-71, Olympus) equipped with a Nipkow disk confocal unit [17], [37], exposed to three different wavelengths of excitation (405, 488 and 561 nm). Images sectioned optically at 2 µm intervals (a total of 100 µm) were acquired in the *z*-axis, and three color images (blue, green and red) were captured. Device control and image analysis were performed using MetaMorph software (Molecular Devices, Sunnyvale, CA, USA).

### Statistical analysis

Outcomes were evaluated using χ^2^ tests, and p<0.05 was regarded as statistically significant.

## Supporting Information

Movie S1 3D clinostat inside CO_2_ incubator
(5.88 MB MOV)

Movie S2 The localization of the ICM in blastocysts using a 3D imaging system (Control 1G)
(9.52 MB MOV)

Movie S3 The localization of the ICM in blastocysts using a 3D imaging system (µG)
(8.86 MB MOV)
